# A cross-sectional study of appropriateness evaluation of anticoagulation therapy for inpatients with nonvalvular atrial fibrillation

**DOI:** 10.3389/fphar.2023.1286559

**Published:** 2023-12-05

**Authors:** Xiao-Yuan Zheng, Guang-Wei Feng, Jing Guo, Fen Xie, Xia Li, Ming-Zhu Zhang, Xiu-Fen Zhang, Xiu-Feng Wu, Yong-Juan Ding

**Affiliations:** ^1^ Department of Pharmacy, Affiliated Hospital of Jiangnan University, Wuxi, China; ^2^ Department of Pharmacy, Dahua Hospital, Xuhui District, Shanghai, China; ^3^ Department of Cardiology, Affiliated Hospital of Jiangnan University, Wuxi, China; ^4^ Department of Pharmacy, Shandong Provincial Third Hospital, Jinan, China; ^5^ Jiangsu Key Laboratory of New Drug Research and Clinical Pharmacy, Xuzhou Medical University, Xuzhou, China; ^6^ Oncology Institute, Affiliated Hospital of Jiangnan University, Wuxi, China

**Keywords:** nonvalvular atrial fibrillation, anticoagulation therapy, non-vitamin K antagonist oral anticoagulants, clinical pharmacist, no-use of OACs

## Abstract

**Background:** Oral anticoagulants (OACs) are essential for stroke prevention in patients with nonvalvular atrial fibrillation (NVAF). However, the appropriateness of anticoagulation treatment in locally practice remains unclear. This study evaluated compliance with anticoagulation therapy concerning the guidelines and drug labels in patients with NVAF.

**Methods:** Hospitalized patients diagnosed with NVAF between 1 November 2020, and 31 December 2021, were retrospectively enrolled. The appropriateness of anticoagulation regimens at discharge was evaluated based on a flowchart designed according to atrial fibrillation (AF) guidelines and medication labels. Furthermore, we explored factors potentially influencing the “no-use of OACs” using binary logistic regression and verified anticoagulation-related issues through a physician questionnaire.

**Results:** A total of 536 patients were enrolled in this study, including 254 patients (47.4%) with inappropriate anticoagulation regimens. 112 patients (20.9%) were categorized as “underdosing-use of OACs,” 134 (25%) who needed anticoagulation therapy were “no-use of OACs” and eight (1.5%) were “over-use of OACs.” The results of a binary logistic regression analysis showed that paroxysmal AF (odds ratio [OR], 7.74; 95% confidence interval [CI], 4.57–13.10), increased blood creatinine levels (OR, 1.88; 95% CI, 1.11–3.16), hospitalized pacemaker implantation (OR, 6.76; 95% CI, 2.67–17.11), percutaneous coronary intervention (OR, 3.35; 95% CI, 1.44–7.80), and an increased HAS-BLED score (OR, 1.62; 95% CI, 1.11–2.35) were associated with “no-use of OACs” in patients with NVAF who had indications for anticoagulation therapy.

**Conclusion:** For patients with NVAF with severe renal dysfunction and paroxysmal AF, anticoagulation therapy was inadequate. The underdosing-use of OACs in patients with NVAF was frequently observed. We recommend an anticoagulation management team to tailor anticoagulation regimens to suit each patient’s needs.

## 1 Introduction

Atrial fibrillation (AF) is a common arrhythmia that becomes more common as people get older; it mostly involves nonvalvular AF (NVAF) (Lippi et al., 2020). Episodes of NVAF are associated with chest tightness, palpitations, progression of heart failure, and even ischemic stroke, which are 4–5 times higher than in those patients without NVAF. Moreover, the incidence of ischemic stroke in Asian patients has increased (Chiang et al., 2017). The 2020 European Society of Cardiology (ESC) guidelines for AF (Hindricks et al., 2021) recommend that men with NVAF and CHA_2_DS_2_-VASc scores ≥2 and women with NVAF and CHA_2_DS_2_-VASc scores ≥3 should be considered for anticoagulation therapy, preferably non-vitamin K antagonist oral anticoagulants (NOACs), to prevent strokes. However, awareness of anticoagulation therapy was inadequate in out-patients with AF, and low-use of OACs was common in practice (Du et al., 2021). Moreover, Asian patients are considered at high risk of hemorrhage and off-label dosage, especially under-dose of NOACs, is regularly prescribed by domestic physicians to patients with AF (Cheng et al., 2019). Poor compliance with guidelines and off-label use of NOACs could cause adverse outcomes (Bang et al., 2023). It is important to highlight compliance with anticoagulation therapy in alignment with guidelines and medication labels in practical clinical settings. This study evaluated the appropriateness of anticoagulation regimens and explored the potential factors associated with inappropriate anticoagulation regimens especially with no-use of OACs locally.

## 2 Materials and methods

### 2.1 Study population

**FIGURE 1:**
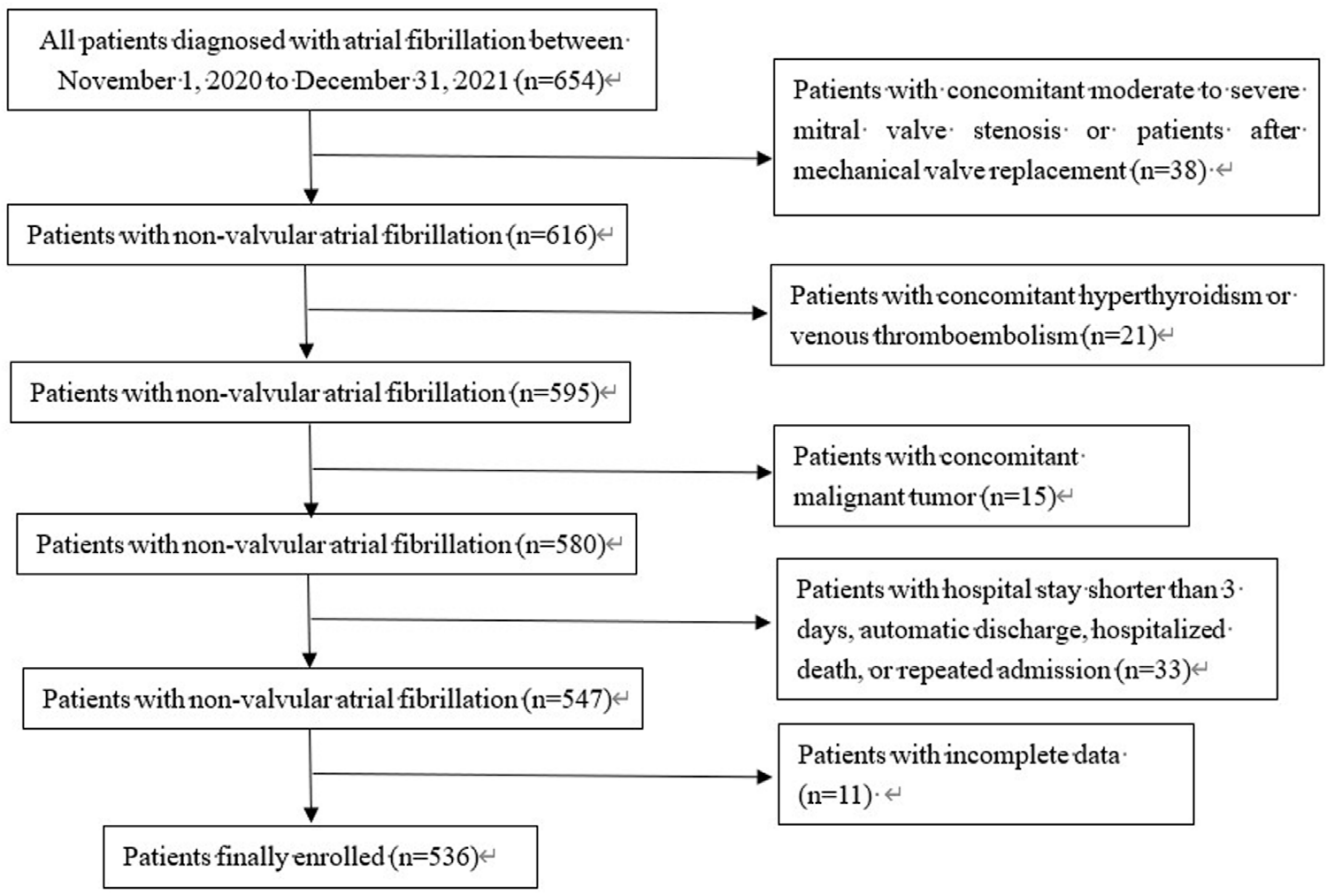
Flowchart of patients enrolled in the study.

All patients diagnosed with AF or atrial flutter admitted to the Affiliated Hospital of Jiangnan University were retrospectively enrolled between 1 November 2020, and 31 December 2021. The inclusion criterion was as follows: diagnosis of AF by ECG within the timeframe. The diagnostic criteria were based on the 2014 American Diagnostic Standards for AF. The exclusion criteria were as follows: patients with moderate to severe mitral stenosis or mechanical valve replacement; hyperthyroidism or malignant tumor; concomitant venous thromboembolism; patients who left the hospital automatically and were transferred to another hospital, those who died, or those who were discharged from the hospital without standardized treatment within 3 hospital-days of admission; those with incomplete data; patients with repeated hospitalization within this period, and patients treated with low-molecular weight heparin (LMWH) at discharge (Figure 1). The Institutional Review Board of the Affiliated Hospital of Jiangnan University approved this study. Written informed consent for participation was not required in this study is in accordance with national legislation and institutional requirements.

### 2.2 Data collection

Demographic data, clinical characteristics, laboratory measurements, and medication use were obtained from the Hospital Information System (HIS). The specific details in the HIS included age, sex, liver and kidney function, platelet counts, coagulation function, concomitant medications, concomitant diseases, and operation type. All patients were scored for their risk of thrombosis and bleeding according to the updated CHA_2_DS_2_-VASc and HAS-BLED scoring systems recommended in the 2020 ESC guidelines for AF.

### 2.3 Outcomes and definitions

**FIGURE 2:**
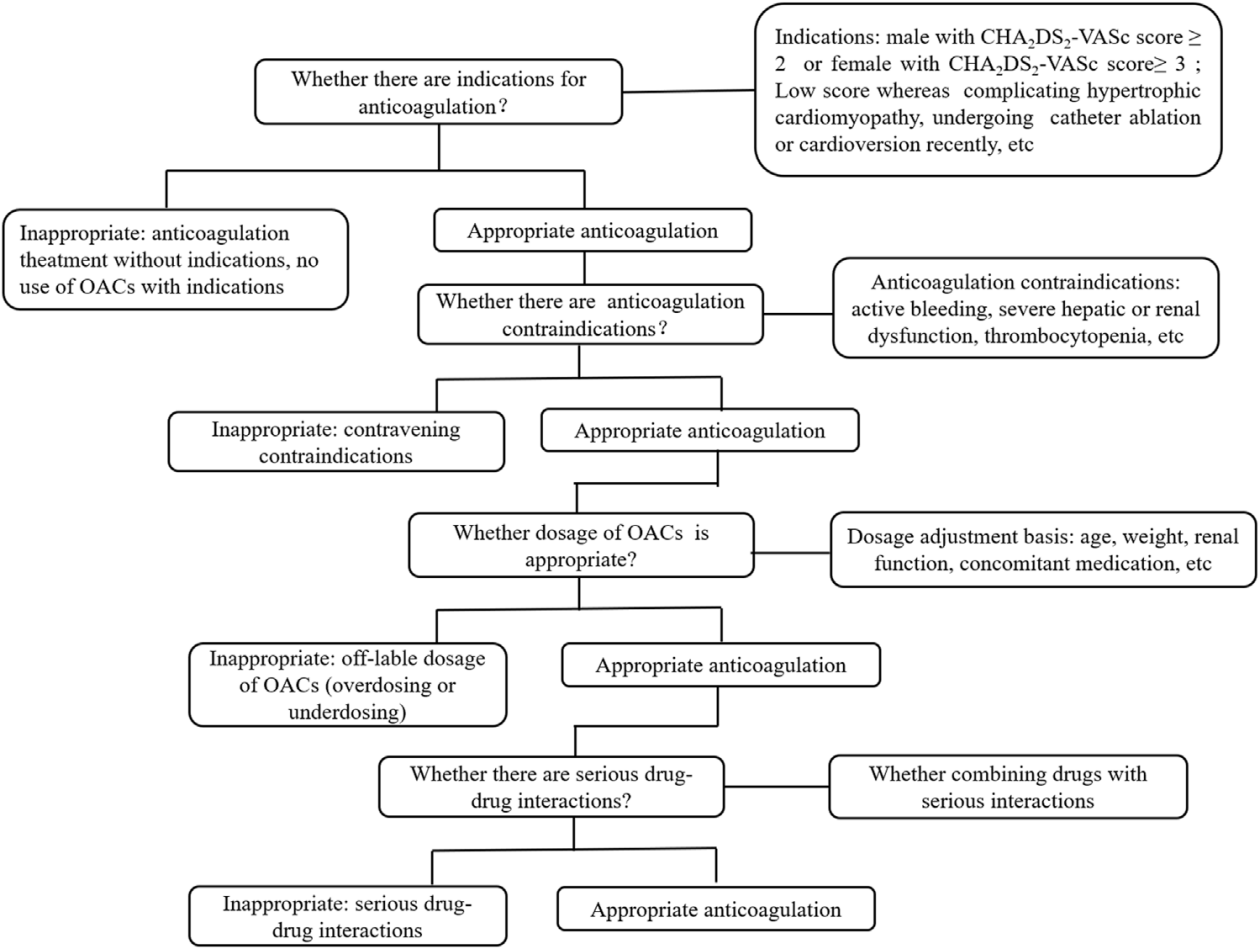
Flowchart of appropriateness assessment of standardized anticoagulation therapy for patients with nonvalvular atrial fibrillation.

Anticoagulation regimens at discharge of all enrolled patients were evaluated according to a flowchart designed basing on the 2020 ESC guidelines for AF, the 2021 European Heart Rhythm Association (EHRA) practical guide on using NOACs in patients with AF, and the drug’s label (Figure 2) (Hindricks et al., 2021; Steffel et al., 2021). The flowchart shows evaluation of anticoagulation regimens, including indications, contraindications, and dosage of oral anticoagulants (OACs). The primary outcomes were as follows: rate of inappropriate anticoagulation therapy consisting of “no-use of OACs,” “underdosing-use of OACs,” “over-use of OACs,” and the factors potentially influencing “no-use of OACs” (Zhao et al., 2022). “No-use of OACs” were defined as patients for whom anticoagulation therapy was indicated and, without contraindications, did not receive OAC treatment. “Underdosing-use of OACs” were defined as patients who did not possess the criterion for reducing the dosage, received a reduced OAC treatment, or who received a smaller OAC dosage not approved by the National Medical Products Administration (NMPA). “Over-use of OACs” were patients who received OAC treatment, but contraindications were breached, and patients for whom anticoagulation therapy was not indicated but still received OAC treatment.

### 2.4 Statistical analyses

Data were collected and input into Excel spreadsheets. Statistical analysis was performed using the SPSS software version 25.0(IBM, Armonk, NY, United States of America). Normally distributed continuous data were presented as the mean ± standard deviation and compared between groups using the Student *t* test. Abnormally distributed continuous data were presented as the median (P_25_, P_75_) and compared between groups using the Mann–Whitney U test. Categorical data were reported as frequencies (%) and compared using the chi-square or the Fisher exact test. A multivariable logistic model included candidate variables with a *p*-value <0.1 by univariate analysis. A binary logistic regression analysis was used to analyze the factors influencing “no-use of OACs.” *p* < 0.05 indicated a statistically significant difference.

### 2.5 Questionnaire design

We developed a ten-question questionnaire to investigate the factors contributing to “no-use of OACs” and other anticoagulation-related concerns in patients with NVAF. The questions focused on the choice of anticoagulation regimen for patients with concomitant paroxysmal AF, renal dysfunction, and other specific conditions that influence anticoagulation therapy. We sent invitations to 14 cardiovascular physicians, asking them to complete the questionnaires, including four chief physicians, four deputy chief physicians, four attending physicians, and two resident physicians (Supplementary Data).

## 3 Results

### 3.1 Population characteristics

In total, 536 patients were enrolled in this study. Of this number, 56.3% of patients were men, 54.9% of patients were aged ≥75 years, 36.0% were diagnosed with paroxysmal AF, and 31.2% were diagnosed with concomitant renal dysfunction. Overall, 91.8% of patients (men with CHA_2_DS_2_-VASc scores ≥2 and women with CHA_2_DS_2_-VASc scores ≥3) had indications for anticoagulation therapy. However, 34.5% did not receive OAC treatment, 14.7% received single antiplatelet therapy (SAPT), 6.9% received dual antiplatelet therapy (DAPT), and 12.9% did not receive antithrombotic treatment (Table 1).

**TABLE 1:** Baseline characteristics of enrolled patients with nonvalvular atrial fibrillation (n = 536).

Variables	Type	N (%)
Age (years)	≤64	83 (15.5)
	65–74	159 (29.7)
	≥75	294 (54.9)
Sex	Women	234 (43.7)
Concomitant disease		
HBP	Yes	390 (72.8)
CHD	Yes	346 (64.6)
PAD	Yes	11 (2.1)
DM	Yes	132 (24.6)
HCM	Yes	10 (1.9)
Heart Function Grade of NYHA	Normal	89 (16.6)
	Class I	34 (6.3)
	Class II	167 (31.2)
	Class III	199 (37.1)
	Class IV	47 (8.8)
Previous stroke/TIA	Yes	104 (19.4)
History of bleeding	Yes	23 (4.3)
Renal dysfunction	Yes	167 (31.2)
Liver dysfunction	Yes	89 (16.6)
AF type	PAF	193 (36.0)
	NPAF	343 (64.0)
CHA_2_DS_2_-VASc score	Men <2 score or Women <3 score	44 (8.2)
	Men ≥2 score or Women ≥3 score	492 (91.8)
HAS-BLED score	<3 score	414 (77.2)
	≥3 score	122 (22.8)
AF catheter ablation	YES	26 (4.9)
Pacemaker implantation	YES	30 (5.6)
CAG	YES	99 (18.5)
PCI	YES	38 (7.1)
Antithrombotic drugs	No medicine	69 (12.9)
	DAPT	37 (6.9)
	DAPT + NOAC	1 (0.2)
	Warfarin	35 (6.5)
	NOAC	295 (55.0)
	SAPT	79 (14.7)
	SAPT + NOAC	20 (3.7)

HBP, high blood pressure; CHD, coronary heart disease; PAD, peripheral arterial disease; DM, diabetes mellitus; HCM, hypertrophic cardiomyopathy; TIA, transient ischemic attack; AF, atrial fibrillation; NYHA, New York Heart Association PAF, paroxysmal atrial fibrillation; NPAF, non-paroxysmal atrial fibrillation; CAG, coronary angiography; PCI, percutaneous coronary intervention; DAPT, dual antiplatelet therapy; NOAC, non-vitamin K antagonist oral anticoagulant; SAPT, single antiplatelet therapy.

### 3.2 Appropriateness of anticoagulation regimens

**FIGURE 3:**
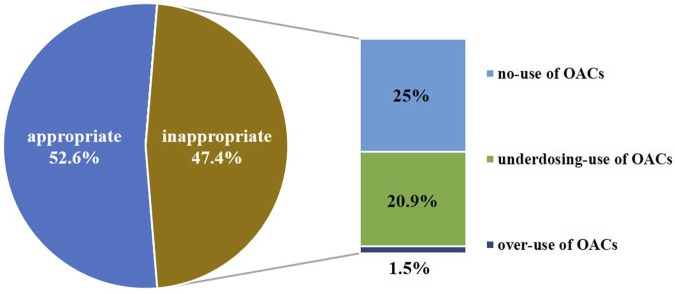
Result of appropriateness assessment of anticoagulation therapy for patients with nonvalvular atrial fibrillation (*n* = 536).

In our evaluation of anticoagulation therapy, of the 536 patients with NVAF, 282 (52.6%) patients received appropriate treatment. Of these 282, 51 patients did not receive OAC treatment because of a low risk of stroke or there were anticoagulation contraindications, and 231patients received appropriate dosage OAC treatment. In contrast, 254 (47.4%) patients received inappropriate treatment. Of them, 134 (25%) patients who needed anticoagulation therapy were categorized as “no-use of OACs,” 112 (20.9%) patients were “underdosing-use of OACs,” and eight patients (1.5%) were “over-use of OACs.” (Figure 3).

### 3.3 Potential factors influencing the no-use of OACs

We analyzed the factors influencing “no-use of OACs” using univariate logistic regression compared with anticoagulation treatment group. These factors included demographic characteristics, concomitant diseases, laboratory monitoring indicators, and hospitalized operation type. Significant variables (*p* < 0.1) were included in the binary logistic regression model and the results showed that paroxysmal AF (OR, 7.74; 95% CI, 4.57–13.10), increased blood creatinine levels (OR, 1.88; 95% CI, 1.11–3.16), hospitalized pacemaker implantation (OR, 6.76; 95% CI, 2.67–17.11), percutaneous coronary intervention (PCI) (OR, 3.35; 95% CI, 1.44–7.80), and an increased HAS-BLED score (OR, 1.62; 95% CI, 1.11–2.35) were associated with “no-use of OACs” patients with NVAF for whom anticoagulation therapy was indicated (Table 2).

**TABLE 2:** Possible factors influencing the no-use of oral anticoagulants for patients with nonvalvular atrial fibrillation (*n* = 477).

Variables	Univariate analysis			Multivariate analysis		
	OR	95% CI	*p-*value	OR	95% CI	*p-*value
Age (years)	1.02	1.00–1.04	0.085	1.02	0.98–1.05	0.314
Men	0.87	0.58–1.29	0.480			
Inpatient days (days)	1.06	1.01–1.11	0.031	1.00	0.94–1.07	0.891
HBP	1.23	0.76–1.98	0.395			
CHD	2.16	1.35–3.43	0.001	1.93	1.00–3.74	0.051
PAD	1.10	0.28–4.32	0.892			
DM	1.60	1.03–2.50	0.038	1.75	0.96–3.17	0.065
HCM	1.29	0.32–5.22	0.725			
Heart Function Grade of NYHA	0.79	0.67–0.94	0.008	0.95	0.75–1.22	0.707
Paroxysmal AF	5.99	3.88–9.25	<0.001	**7.74**	**4.57–13.10**	**<0.001**
Previous stroke/TIA	1.09	0.66–1.78	0.738			
History of bleeding	1.10	0.28–4.32	0.892			
Blood creatinine level	1.78	1.15–2.76	0.010	**1.88**	**1.11–3.16**	**0.018**
Liver dysfunction	0.57	0.32–1.02	0.060	0.84	0.41–1.72	0.626
CHA_2_DS_2_-VASc score	1.13	1.00–1.27	0.047	0.82	0.62–1.08	0.159
HAS-BLED score	1.47	1.18–1.83	<0.001	**1.62**	**1.11–2.35**	**0.012**
AF catheter ablation	0.10	0.01–0.75	0.025	**0.09**	**0.01–0.69**	**0.021**
CAG	0.87	0.52–1.45	0.585			
PCI	3.80	1.92–7.53	<0.001	**3.35**	**1.44–7.80**	**0.005**
Pacemaker implantation	4.99	2.30–10.79	<0.001	**6.76**	**2.67–17.11**	**<0.001**

The bold values indicate statistical significance (*p* < 0.05).

AF, atrial fibrillation; HBP, high blood pressure; CHD, coronary heart disease; PAD, peripheral arterial disease; DM, diabetes mellitus; HCM, hypertrophic cardiomyopathy; NYHA, new york heart association; TIA, transient ischemic attack; CAG, coronary arteriography; PCI, percutaneous coronary intervention; CI, confidence interval; OR, odds ratio.

### 3.4 Findings from questionnaire for physicians

The following findings were obtained from the physician questionnaire (Supplementary Data):

Question 1: Approximately 50% of physicians restarted NOACs at least 1 week after pacemaker implantation.

Question 2–3: Physicians partially discontinued long-term anticoagulation therapy in patients with paroxysmal AF after successful cardioversion to sinus rhythm using amiodarone or catheter ablation.

Question 4: Most physicians overestimated HAS-BLED scores in clinical practice.

Question 5–6: Most physicians were reluctant to prescribe warfarin to patients with severe renal dysfunction (CrCl <30 mL/min) or tended to prescribe low-dose NOACs or discontinued anticoagulation therapy altogether.

Question 7–8: For patients with paroxysmal AF undergoing PCI, a small number of physicians tended toward DAPT.

Question 9: Fifty percent (50%) of physicians tended toward SAPT instead of anticoagulation therapy in patients with end-stage renal disease (ESRD) and concomitant stable coronary heart disease (CHD).

Question 10: Underdosing-use of NOACs was commonplace in clinical practice.

## 4 Discussion

OACs, particularly NOACs, are essential for preventing stroke in patients with NVAF. However, the appropriateness of anticoagulation treatment in locally practice remains unclear. In this cross-sectional retrospective study, clinical pharmacists evaluated 536 AF inpatient records examining anticoagulation regimens based on the latest guidelines and medication labels. Similar studies also evaluated the appropriateness of OAC treatment in patients with AF (Qian et al., 2021; Delesie et al., 2022; Moudallel et al., 2022) and explored the possible influencing factors regarding inappropriate anticoagulation therapy. Distinct from those studies, our study designed, produced, and disseminated a questionnaire and asked the “no-use of OACs” to verify the influencing factors. As a result, we designed a flowchart for administering individualized anticoagulation regimens for patients diagnosed with NVAF. These measures aimed to improve therapeutic quality and involve clinical pharmacists in anticoagulation management.

The study showed approximately 47.4% (254/536) of the anticoagulation regimens for AF patients upon discharge were inappropriate. The inappropriate sample consisted of 134 (25%) “no-use of OACs”, 112 (20.9%) “underdosing-use of OACs”, and eight (1.5%) “over-use of OACs”. Comparisons between the “no-use of OACs” and anticoagulation treatment group showed that paroxysmal AF, increased blood creatinine levels, hospitalized pacemaker implantation, PCI, and increased HAS-BLED scores were associated with no-use of OAC treatment. Furthermore, underdosing-use of OACs in patients with NVAF was commonplace in locally practice.

### 4.1 “Paroxysmal AF” leading to no-use of OACs

Among patients with NVAF, those with paroxysmal AF were frequent, including those newly diagnosed and those who had undergone catheter ablation. The stroke risk associated with paroxysmal AF appears lower than with persistent AF (Genovesi-Ebert et al., 2020). However, the risk factors for stroke in patients with paroxysmal AF are usually less than those in patients with persistent AF. In other words, the CHA_2_DS_2_-VASc score of patients with paroxysmal AF is routinely lower than those with persistent AF. The 2020 ESC guidelines for AF recommend anticoagulation therapy depending on the CHA_2_DS_2_-VASc score rather than the type of AF. Patients with AF and high CHA_2_DS_2_-VASc scores need to be treated with anticoagulation therapy to prevent stroke or systemic embolism, even if they are diagnosed with paroxysmal AF or have been treated with catheter ablation. In clinical practice, physicians are reluctant to prescribe OACs to patients with paroxysmal AF, especially 3 months after catheter ablation. A US national registry involving 21,595 patients with AF showed that 23% of patients discontinued OAC treatment after catheter ablation despite a high stroke risk score (Freeman et al., 2019). OAC discontinuation after catheter ablation was controversial because the evidence was limited to observational data. While the definition of “successful ablation” remains ambiguous, maintaining sinus rhythm to reduce the incidence of stroke was uncertain (Chew and Piccini, 2021). Anticoagulation therapy should be administered to patients with paroxysmal AF who do not undergo catheter ablation and have a high CHA_2_DS_2_-VASc score.

### 4.2 “Increased blood creatinine levels” leading to no-use of OACs

The study revealed that severe renal dysfunction was an independent factor associated with the no-use of OACs in patients with NVAF. Some patients have AF and concomitant chronic kidney disease (CKD) due to their shared risk factors, including hypertension, diabetes mellitus, and coronary artery disease (Ding et al., 2021). Previous studies have revealed that renal dysfunction is an independent risk factor for new-onset AF. Patients with a CrCl <60 mL/min or chronic renal dysfunction together with proteinuria have a higher prevalence of AF than healthy individuals (Guo et al., 2019; Kodani et al., 2020). Furthermore, patients with AF are an increasingly aging population with a gradual decline in renal function. Long-term AF causes a decline in cardiac function, ultimately leading to reduced renal perfusion and further damage (Carlisle et al., 2019). Patients with AF and renal dysfunction have an increased risk of thrombosis and bleeding (Parker et al., 2022). Anticoagulation therapy is challenging for these patients, particularly in patients with ESRD, because NOACs partly depend on renal clearance. Although warfarin was approved for domestic use in patients with concomitant ESRD and AF, physicians were reluctant to prescribe warfarin because of the need for frequent monitoring of the international normalized ratio (INR). Furthermore, the benefits and bleeding risks associated with warfarin were controversial for patients with CrCl <15 mL/min (Ha et al., 2019). However, for patients with AF and mild to moderate renal dysfunction, NOACs have been associated with a lower risk of acute kidney injury, CKD progression, and a better clinical profile of net clinical benefit (Gu et al., 2019; Trevisan et al., 2023). Some physicians in practice did not prescribe NOACs due to concerns about hemorrhage, even for patients with moderate renal dysfunction, let alone patients with severe renal dysfunction. Physicians were concerned that patients’ renal function was over-assessed by different methods, such as Modification of Diet in Renal Disease (MDRD), Chronic Kidney Disease Epidemiology Collaboration (CKD-EPI), and Cockcroft-Gault (CG), and other methods showing inconsistencies in assessing renal dysfunction (Rohla et al., 2021). Anticoagulation management requires intensive follow-up to monitor renal function for the dynamic assessment of bleeding risk. However, domestic follow-up is inadequate due to the limited time and effort of domestic physicians.

### 4.3 “Hospitalized pacemaker implantation” leading to no-use of OACs

The 2021 EHRA practical guide to using NOAC in patients with AF stated that coronary intervention and pacemaker implantation were both invasive procedures with minimal risk of bleeding, and the NOAC regimens can be resumed 24 h after operation in patients with normal renal function. In a real-world setting, some physicians did not administer NOACs within 7 days of pacemaker implantation, despite the patients’ normal renal function, because they believed early NOACs might lead to pacemaker pocket hematoma. The BRUISE CONTROL study (Birnie et al., 2013) showed that uninterrupted warfarin administration during the perioperative period of pacemaker implantation can significantly reduce the incidence of hematoma and hospital stay compared with low-molecular-weight heparin. Subsequently, the BRUISE CONTROL-2 study (Birnie et al., 2018) from 2013–2017 aimed to explore whether continuous treatment with NOACs could reduce the incidence of pocket hematoma after pacemaker implantation. However, the study was halted because the sample size needed to be enlarged following mid-term data analysis. In other words, present studies have found no significant correlation between NOAC interruption and the incidence of pocket hematoma. However, discontinuation of NOACs increase the risk of ischemic stroke. Therefore, the perioperative anticoagulation management of pacemaker implantation, which involves the pacemaker type, surgical method, personal platelet function, and thrombotic risk, needs further exploration. Multidisciplinary team involvement is necessary for the perioperative management of patients with NVAF, and early restoration of anticoagulation therapy is essential in patients with NVAF with a high risk of stroke.

### 4.4 “PCI” leading to no-use of OACs

This study found that three-quarters of patients with NVAF with no-use of OACs received antiplatelet treatment (SAPT or DAPT). Moreover, results of a binary logistic regression analysis indicated that PCI was associated with no-use of OACs. Previous studies had showed that OACs can more effectively reduce the incidence of stroke in patients with NVAF than antiplatelet drugs (Hohnloser et al., 2007). However, in practice, a small proportion of physicians and patients tend to use antiplatelet drugs to prevent stroke in patients with NVAF to replace OACs, particularly in those with concomitant CHD. Antiplatelet drugs are seemingly safer and are more commonly used than OACs in clinical practice. Nevertheless, antiplatelet and anticoagulation therapies may have similar bleeding risks, depending only on the hemorrhage risk factors in patients (Olesen et al., 2011). From an analysis of physicians’ medication habits, this phenomenon was obvious in patients with AF who underwent PCI and was attributed to DAPT treatment after PCI. Nevertheless, the 2021 EHRA practical guide on using NOACs in patients with AF and the 2020 ESC guidelines for AF stated that OACs should be consistently prescribed to patients with AF with concomitant CHD, whether or not these patients undergo PCI due to acute coronary syndrome (ACS) or chronic coronary syndrome (CCS). Patients were selectively treated with DAPT or SAPT and OACs based on a comprehensive assessment of the risk of thrombosis and bleeding. This work may require time and effort to weigh the risks, and the involvement of a multidisciplinary team in shared decision-making principles is recommended.

### 4.5 “High HAS-BLED score” leading to no-use of OACs

The HAS-BLED score is widely used in clinical practice as an assessment tool for the risk of bleeding in patients with AF. This score was proposed and validated in an early cohort study (Pisters et al., 2010), wherein “A” was defined as liver or renal dysfunction, including the presence of chronic dialysis, renal transplantation, or serum creatinine >200 μmol/L. In practice settings, most physicians considered all types of renal dysfunction as bleeding risk factors. Accordingly, the bleeding risk score was overestimated. Similarly, hypertension was also listed in the HAS-BLED score even if the patient’s blood pressure was well-controlled. A higher HAS-BLED score that does not support using OACs is reasonable. However, improper understanding of the HAS-BLED score may be an obstacle to anticoagulation therapy.

### 4.6 Underdosing-use of OACs

In our study, 59.0% of all enrolled patients (536) were prescribed NOACs, including rivaroxaban, dabigatran, and edoxaban. Apixaban was excluded, which was not approved in patients with NVAF by the NMPA in China. Moreover, only 6.5% of patients were prescribed warfarin. Compared with the average domestic level, the total use of NOACs was significantly high (Du et al., 2021). However, nearly half of the NOAC dosages were off-label, mainly because of underdosing. The standard dosage of rivaroxaban is 20 mg once daily, and that of dabigatran is 150 mg twice daily in patients with NVAF for stroke prevention. Additionally, the dosages of NOACs can be adjusted according to a comprehensive assessment of patients’ weight, age, renal function, and concomitant medications. Detailed principles of use have been individually documented for every medication label approved by the NMPA in China. However, underdosing-use of NOACs was commonplace in practice. For example, the usual dose of rivaroxaban prescribed by physicians was 15 mg or even 10 mg once daily, which was an off-label dosage.

Several studies had confirmed that underdosing-use of NOACs was associated with an increased risk of all-cause mortality or thrombotic events (Steinberg et al., 2016; Nielsen et al., 2017; Yao et al., 2017; Camm et al., 2020; Lee et al., 2020). Out of these studies, the most recent one focused on Asian patients but was conducted in a single center. It found that using a lower dose of NOAC (which was an off-label use) was linked to a 2.5-times higher risk of thromboembolism compared with using warfarin (Lee et al., 2020). Additionally, in a study conducted by Xu et al., in 2023, it was reported that the off-label use of underdosed NOACs in Asian patients with AF was associated with significantly reduced risks of major bleeding and all-cause mortality when compared with the recommended dosage. Although this study and Lee et al., 2020 provided evidence for the off-label use of NOACs, both studies may be influenced by confounders due to their retrospective nature. Furthermore, in the former study by Lee et al., 2020, the under dosage of NOACs was compared with warfarin, and in the latter study by Xu et al., 2023, it was compared with the recommended dosage of NOACs. We conclude that off-label dosage of NOACs remains a matter of debate for the Asian population. The off-label use of these medications requires a comprehensive prospective study involving a substantial number of Asian patients with AF to validate its effectiveness, particularly since it has not yet received approval from the NMPA, at least as of now. Therefore, NOACs should be individually prescribed. In particular, patients with a high stroke risk and low bleeding risk should be treated with the full dose.

### 4.7 Questionnaire

The questionnaire aimed to investigate physicians’ understanding of anticoagulation therapy, as this knowledge influenced their selection of anticoagulation plans for patients with NVAF. The questionnaire was designed to address the gap between practice and guidelines. According to the questionnaire, we discovered a change in physicians’ choice of anticoagulation regimen in patients with NVAF during the perioperative period of PCI. The questionnaire was administered 1 year after data collection of this study. For patients with persistent AF undergoing PCI due to acute coronary syndrome, 57% of physicians chose triple therapy with aspirin, clopidogrel, and NOAC for 1 month after PCI, and 43% of physicians chose dual therapy with clopidogrel and NOAC. Both regimens involved using OACs. Among the ten questions on anticoagulation in patients with NVAF, each question contained an option for SAPT. However, almost none of the physicians chose SAPT in the first nine questions. SAPT was chosen only for patients with ESRD (10 mL/min < CrCl <15 mL/min) and concomitant CHD. Some physicians tended toward SAPT, which was approved in patients with ESRD when using OACs was challenging. This indicated that nowadays, almost all physicians in our hospital regard anticoagulation therapy as the first-line treatment for patients with NVAF, along with various updated guidelines, and antiplatelet therapy as secondary replacement therapy in special cases. However, a few physicians chose the DAPT regimen consisting of aspirin and clopidogrel without OAC after PCI for patients with CCS and concomitant paroxysmal AF. Despite patients having a CHA_2_DS_2_-VASc score of ≥3, physicians were reluctant to prescribe OACs for paroxysmal AF. In other words, physicians considered the stroke risk of patients with paroxysmal AF low. This awareness was verified using multiple questions in the questionnaire. Fifty percent of physicians would discontinue anticoagulation treatment after successful cardioversion by drugs or catheter ablation for patients with NVAF, especially for patients with rare episodes of AF after catheter ablation.

### 4.8 What pharmaceutical care can we provide?

In conclusion, there are some difficulties standardizing anticoagulation treatment, which is essential for patients with NVAF. In clinical practice, we intend to intervene to improve guideline-adherence for anticoagulation therapy. Since managing patients with AF is complex, an anticoagulation team, including physicians, clinical pharmacists, and nurses from multiple disciplines, is necessary (Gebreyohannes et al., 2021; Koolian et al., 2022). Subsequently, for patients diagnosed with NVAF, the team could administer individualized anticoagulation regimens based on the patient’s age, liver function, kidney function, concomitant disease, concomitant drugs, and operation type, according to shared decision-making principles (Supplementary Table S1 and Supplementary Figure S1). Many hospitals in China have implemented the Physician-Pharmacist Collaborative Clinic and have achieved promising results (Wang et al., 2021). Furthermore, genetic testing and blood medication concentration testing to guide individualized medication choice of NOACs is a promise direction being leaded mostly by clinical pharmacists in an anticoagulation team (Gu et al., 2018).

Additionally, clinical pharmacists can provide anticoagulation education to patients using various tools, such as flyers, brochures, and posters. Medication education can establish awareness of anticoagulation treatment and enhance medication adherence in patients with AF. Pharmaceutical services can improve the safety and efficacy of medications, such as OACs high-alert drugs (Khan et al., 2022). Furthermore, clinical pharmacists can also complete follow-ups for patients with AF using powerful follow-up software to ensure correct adherence to medication, which is important for treating chronic diseases (Zhang et al., 2021). In particular, this study showed that many patients with concomitant AF and renal dysfunction required intensive follow-up after anticoagulation therapy. In addition, adverse drug-induced events can be efficiently reduced by timely rectification of incorrect dosages and drug combinations during follow-up.

### 4.9 Limitations

This study had some limitations. First, the cohort included only patients with AF admitted to a cardiology department in a single hospital within a specified period, and the population was selectively biased. Second, the study lacked patient follow-up after discharge to determine medication compliance. Therefore, the usage rate of OACs in practice may have been overestimated. Third, this study did not analyze patients’ anticoagulation awareness, financial status, and educational background, which may have influenced the use of OACs. Fourth, only a small number of physicians were invited to participate in the questionnaire, and a large-scale questionnaire involving multicenter should be collected to confirm our results, especially for the overestimated HAS-BLED scores. Fifth, apixaban was not domestically approved and was excluded from this study, so that this study cannot reflect all NOACs use.

## 5 Conclusion

For patients with NVAF with severe renal dysfunction and paroxysmal AF, anticoagulation therapy was insufficient. Underdosing-use of NOACs in patients with NVAF is common in practice. Individualized anticoagulation regimens should be administered by an anticoagulation management team (including physicians, clinical pharmacists, and nurses from multiple disciplines) based on the patient’s age, liver function, kidney function, concomitant disease, concomitant drugs, and operation types, according to the shared decision-making principles.

## Data Availability

The original contributions presented in the study are included in the article/Supplementary Material, further inquiries can be directed to the corresponding author.
