# Supplementary material for: A cross-sectional study of appropriateness evaluation of anticoagulation therapy for inpatients with nonvalvular atrial fibrillation
Source: Front Pharmacol. 2023 Dec 5;14:1286559. doi: 10.3389/fphar.2023.1286559 (PMC10728771; doi:10.3389/fphar.2023.1286559)
Supplement: Supplementary file 1 [file Table1.DOCX]

Supplementary Material

# Supplementary Data:

# Questionnaire on anticoagulation-related problems in patients with nonvalvular atrial fibrillation

1. Patients received rivaroxaban for a long time to prevent stroke, and “pacemaker implantation” will be performed to discontinue rivaroxaban. How long do you prefer to start anticoagulation therapy after the operation, provided that the renal function is normal?
2. For patients with paroxysmal atrial fibrillation with a CHA_2_DS_2_-VASc score of 3 (after admission, atrial fibrillation rhythm turned into sinus rhythm using “amiodarone”), which of the following options would you tend toward after discharge?
3. For patients with paroxysmal atrial fibrillation with a CHA_2_DS_2_-VASc score of 3 (who have undergone catheter ablation after admission), which of the following options would you tend toward after discharge?
4. What is the HAS-BLED score of the patient with the following status: 76 years old, Grade 3 hypertension (well-controlled blood pressure), type 2 diabetes, persistent atrial fibrillation, and chronic renal disease (serum creatinine, 150 μmol/ L)?
5. For patients with persistent atrial fibrillation and renal dysfunction (CrCl, 12 mL/min) without dialysis who require anticoagulation treatment, which of the following options would you tend toward?
6. For patients with persistent atrial fibrillation and renal dysfunction (CrCl, 25 mL/min) who require anticoagulation treatment, which of the following options would you tend toward?
7. For patients with persistent atrial fibrillation undergoing “percutaneous coronary intervention” owing to acute coronary syndrome, which of the following options would you tend toward after discharge?
8. For patients with paroxysmal atrial fibrillation undergoing “percutaneous coronary intervention” owing to chronic coronary syndrome, which of the following options would you tend toward after discharge?
9. For patients with atrial fibrillation, chronic kidney disease (CrCl, 10-15 mL/min), and stable coronary heart disease who are at a high risk of stroke and bleeding simultaneously, which of the following options would you tend toward? (multiple choice permissible).
10. Which dosage would you choose for a patient with nonvalvular atrial fibrillation having the following status: male; 65 years old; weighing 75 kg; CHA_2_DS_2_-VASc score of 5; HAS-BLED score of 1; possessing normal renal function; taking release-controlled nifedipine, valsartan, furosemide, and spironolactone as a usual? (multiple choice permissible).

# Supplementary Figure and Table:

**Supplementary Table 1.** Contraindications and dosages of anticoagulants approved by the National Medical Products Administration for patients with nonvalvular atrial fibrillation

| Drug | Contraindication | Standard dosage and the principle of dose adjustment |
| --- | --- | --- |
| Warfarin | Bleeding or bleeding tendency (active bleeding, cerebral aneurysm, aortic dissection aneurysm, pericarditis, pericardial effusion, infective endocarditis, thrombocytopenia) or cachexia; recent or expected central nervous system and eye surgery; spinal puncture; allergies to warfarin or any other component of the product; severe liver dysfunction and cirrhosis; malignant hypertension. | Adjust dosage according to the international normalized ratio. |
| Dabigatran | Patients with known allergy to active ingredients or any excipient of the product; patients with severe renal dysfunction (CrCl < 30mL/min); lesions or conditions with significant risk of massive hemorrhage; concomitant use of other anticoagulants; concomitant liver dysfunction affecting the survival time; concomitant use of cyclosporin, systemic ketoconazole, itraconazole and dronedarone; after mechanical heart valve replacement | 1. Standard dosage: 150 mg twice daily.  2. The criteria for reducing dosage to 110 mg twice daily is as follows: age ≥ 75 years; the creatinine clearance rate maintained at 30-50 mL/min; patients with concomitant antiplatelet therapy or non-steroidal anti-inflammatory drugs; patients with previous gastrointestinal bleeding, gastritis, esophagitis requiring use of antacid drugs; weight < 50 kg; concomitant use of moderate P-gp inhibitors such as amiodarone, verapamil, and quinidine. |
| Rivaroxaban | Patients allergic to rivaroxaban or any excipient in tablets; patients with clinically significant active bleeding; lesions or conditions with significant risk of massive hemorrhage; concomitant use of other anticoagulants; patients with liver disease leading to abnormal coagulation function and clinically related bleeding risk, including patients with liver cirrhosis (Child Pugh class B or C); concomitant use of powerful P-gp and CYP3A4 inhibitors such as dronedarone, ketoconazole, and ritonavir. | 1. Standard dosage: 20 mg once daily.  2. The criteria for reducing dosage to 15 mg once daily is as follows: age ≥ 75 years; the creatinine clearance rate maintained at 15-49 mL/min; weight < 50 kg; concomitant use of moderate P-gp inhibitors and CYP3A4 inhibitors such as erythromycin, clarithromycin, and amiodarone. |
| Edoxaban | Patients allergic to this product or other auxiliary materials; patients with clinically significant active bleeding; patients with liver disease leading to coagulation dysfunction and clinically related bleeding risk; lesions or conditions with significant risk of massive hemorrhage; uncontrolled severe hypertension; pregnant and lactating women. | 1. Standard dose: 60 mg qd.  2. The criteria for adjusting the dosage to 30 mg qd: body weight ≤ 60 kg; the glomerular filtration rate of 15-50 mL/min; combined use of P-gp inhibitors such as cyclosporine, dronedarone, erythromycin, ketoconazole, quinidine, and verapamil. |


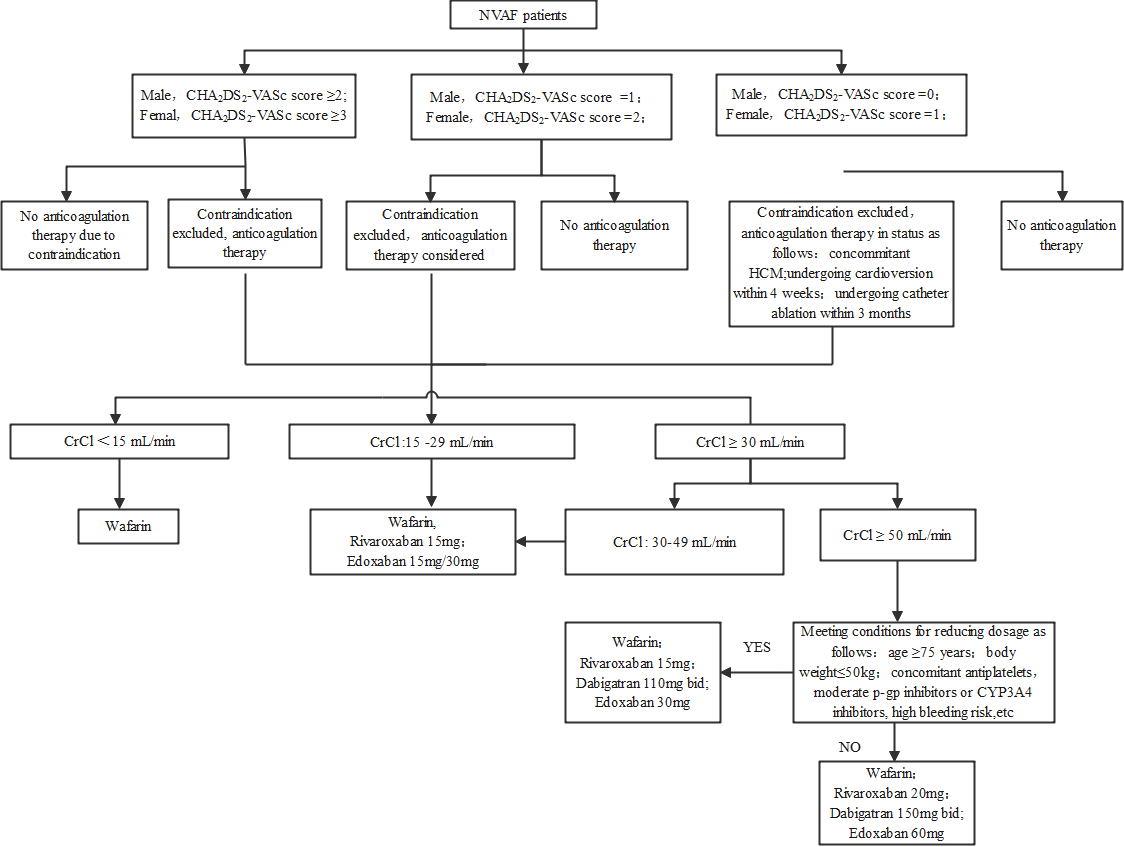


**Supplementary Figure 1.** Flowchart of the standardized anticoagulation therapy in patients with nonvalvular atrial fibrillation

NVAF, nonvalvular atrial fibrillation; HCM, hypertrophic cardiomyopathy
